# Gastrointestinal disorders as immune-related adverse events

**DOI:** 10.37349/etat.2021.00039

**Published:** 2021-04-30

**Authors:** Daniele Balducci, Claudia Quatraccioni, Antonio Benedetti, Marco Marzioni, Luca Maroni

**Affiliations:** Clinic of Gastroenterology and Hepatology, Università Politecnica delle Marche, Ospedali Riuniti-University Hospital, 60126 Ancona, Italy; University of Southampton, UK

**Keywords:** Cytotoxic T-lymphocyte antigen 4, programmed cell death 1, programmed cell death-ligand 1, immune checkpoint, gastrointestinal toxicity

## Abstract

Immune checkpoint inhibitors, such as cytotoxic T-lymphocyte antigen 4 inhibitors, programmed cell death 1 inhibitors and programmed cell death-ligand 1 inhibitors, have recently emerged as novel drugs in the anti-cancer therapy. Their use in different types of advanced cancer has shown good results and an increase in survival rates. However, immune-related adverse events (irAEs) are frequent and often require special care. IrAEs may affect all the organs, but they are most commonly seen in skin, lungs, endocrine glands and in the gastrointestinal tract where small bowel, colon, the liver and/or the pancreas can be involved. Despite being usually mild and self-resolving, irAEs may present in severe and life-threatening forms, causing the withdrawal of anti-cancer therapy. IrAEs, therefore, represent a challenging condition to manage that often requires the cooperation between the oncologists and the gastroenterologists in order to identify and treat them adequately.

## Introduction

Immune checkpoint inhibitors (ICIs), including anti-cytotoxic T-lymphocyte antigen 4 (CTLA-4) antibodies and agents directed against the programmed cell death 1 (PD-1)/programmed death ligand 1 (PD-L1), have demonstrated to be effective in the treatment of numerous cancers, including advanced melanoma, non-small and small lung cancer, renal carcinoma and hepatocellular carcinoma among many others [1]. Despite the overall increase in survival rate of patients with advanced cancers, immune-related adverse events (irAEs) are not rare. IrAEs can manifest as mild self-limiting symptoms, but severe life-threatening events are also reported in the literature. In severe cases specific treatment and permanent immunotherapy discontinuation are required. Despite all systems may be affected, irAEs predominantly involve the skin and the gastrointestinal (GI) tract [2].

## Mechanism of action of ICIs

Naive T helper cells are activated when the interaction between the T cell receptor (TCR) and its the major histocompatibility complex (MHC) II-peptide complex is followed by the binding of the T cell costimulatory receptors to its ligand on the antigen-presenting cell (APC) [3, 4]. The absence of the “second signal” prevents the mounting of a T cells response [5]. The costimulatory receptor CD28 binds to two ligands: CD80 and CD86 (B7 ligands), both expressed by APCs [6]. In order to limit overstimulation of the immune response and maintain self-tolerance, inhibitory receptors (also called immune checkpoints) such as CTLA-4, PD-1 and others, are also expressed during T cell activation. The stimulation of immune checkpoints results in suppression of the immune system. A number of studies have shown that these inhibitory signaling pathways are involved in tumor surveillance since they may hamper the immune response against cancer [7].

**Figure 1.**
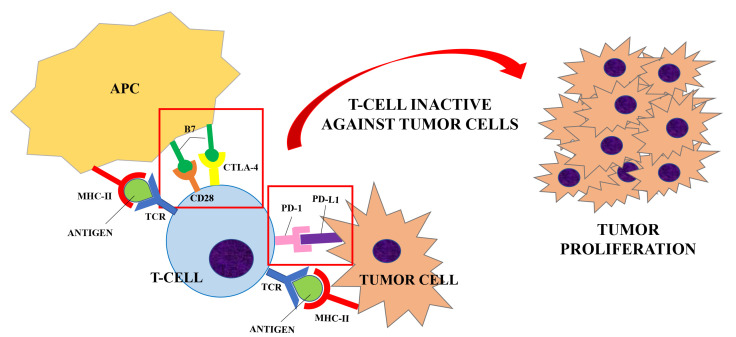
Inhibitory receptors (or immune checkpoints), such as CTLA-4 or PD-1, limit the overstimulation of the immune system during its activation. CTLA-4 is expressed on regulatory T-cells and competes with CD28 for the B7 ligand (CD80 and CD86) expressed by APCs. PD-1 is expressed by T-cells, B-cells and NK cells and it binds to PD-L1, expressed by tumor cells. When T-cell are activated (upon the interaction of TCR and MHC-II peptide complex), the interaction between CTLA-4/PD-1 and its ligand results in the suppression of the immune system. A number of studies have shown that these inhibitory signaling pathways are involved in tumor surveillance since they may hamper the immune response against cancer [7], causing a proliferation of tumor cells

**Figure 2.**
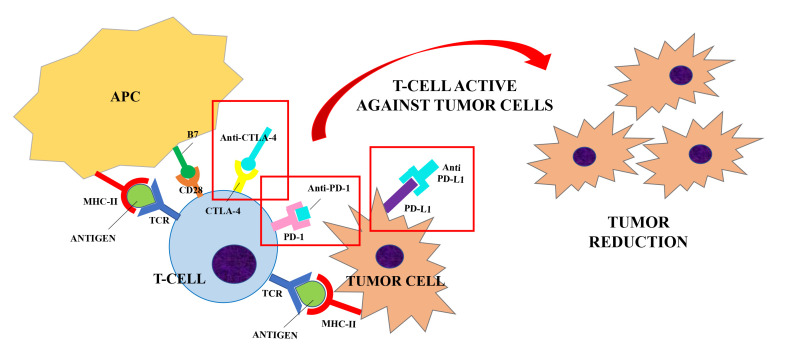
ICIs (Anti-CTLA-4, Anti-PD-1 and Anti-PD-L1) are developed to block the interaction between the inhibitory receptors (CTLA-4, PD-1) and their ligands (B7, PD-L1) to unbreak the T-cells response against tumor cells. Anti-CTLA-4/CTLA-4 interaction increases the CD28 co-stimulatory activity and induces a strong activation of the T-cells against tumor cells. Similarly, anti-PD-1 and anti-PD-L1 were shown to enhance T cells anti-tumor response. Their use in clinical trials has resulted in improved survival for adults with advanced tumors

CTLA-4 is constitutively expressed on regulatory T cells (Treg) and is induced in activated Foxp3^neg^ CD4^+^ T and CD8^+^ T cells. CTLA-4 competes with CD28 for the same ligands (CD80 and CD86), producing a negative signal which results in an inhibition of interleukin (IL)-2 production and T-cell proliferation [8]. Preliminary evidence suggests that CTLA-4 modulation can “unbreak” the cytotoxic T cell response. The proposed mechanism of action (Figures 1 and 2) consists of both an indirect effect through inhibition of Treg accumulation in tumor and a direct cytotoxic T lymphocytes action through inhibition of CTLA-4 interaction with B7 ligands. The result is an increasing CD28 co-stimulatory activity and a more effective immune response against tumor cells [9, 10].

PD-1 is expressed by B cells, T cells and natural killer (NK) cells upon activation and transmits inhibitory signals when activated by its ligands, i.e. PD-L1 and PD-L2. PD-L1 is expressed by tumor cells, immune cells and endothelial cells after cytokine activation, whereas PD-L2 is present only in dendritic cells in normal tissue [11, 12]. PD-1 becomes clustered with the TCR upon binding to its ligand PD-L1 and induces the dephosphorylation of the proximal TCR signaling molecules. This results in the suppression of T cell activation [13]. Antibody blockade of PD-1 was shown to enhance T cell anti-tumor response, supporting its rational for cancer immunotherapy [14], but with milder adverse events (AEs) than CTLA-4 blockade [15].

Monoclonal antibodies that can exploit these targets have been developed during the last decade. Their use in clinical trials has resulted in improved survival and durable response rates for adults with advanced tumors [11, 15]. Therefore, ICIs have been approved for the treatment of several diseases, including melanoma, non-small cell lung cancer and renal cell carcinoma. Currently Food and Drug Administration (FDA)-approved ICIs include: (1) the anti-CTLA-4 agents ipilimumab; (2) the anti-PD-1 agents nivolumab, pembrolizumab, cemiplimab; (3) the anti-PD-L1 inhibitors atezolizumab, avelumab and durvalumab [1]. The same drugs are approved by European Medicines Agency (EMA) with some different indications which are summarized in Table 1 [16–22].

**Table 1.** ICIs indications approved by FDA and EMA

**Drug**	**FDA indications**	**EMA indications**
Ipilimumab	Melanoma Non-small cell lung cancer Renal cell carcinoma	Melanoma Non-small cell lung cancer Renal cell carcinoma data
Nivolumab	Melanoma Non-small cell lung cancer Small cell lung cancer Renal cell cancer Classical Hodgkin’s lymphoma Squamous cell of esophagus or head and neck Urothelial cancer Colorectal cancer with microsatellite instability or mismatch-repair deficiency Hepatocellular carcinoma	Melanoma Non-small cell lung cancer Renal cell cancer Classical Hodgkin’s lymphoma Squamous cell of esophagus or head and neck Urothelial cancer
Pembrolizumab	Melanoma Non-small cell lung cancer Squamous cell of the head and neck Classical Hodgkin’s lymphoma Urothelial cancer Gastric cancer Esophageal cancer Cervical cancer Renal cell carcinoma Hepatocellular carcinoma Colorectal cancer with microsatellite instability or mismatch-repair deficiency	Melanoma Non-small cell lung cancer Squamous cell of the head and neck Classical Hodgkin’s lymphoma Urothelial cancer Colorectal cancer with microsatellite instability or mismatch-repair deficiency
Cemiplimab	Cutaneous cell carcinoma	Cutaneous cell carcinoma
Atezolizumab	Urothelial carcinoma Non-small cell lung cancer Small-cell lung cancer Breast cancer Hepatocellular carcinoma	Urothelial carcinoma Non-small cell lung cancer Small-cell lung cancer Breast cancer Hepatocellular carcinoma
Avelumab	Merkel cell carcinoma Urothelial carcinoma Renal Cell carcinoma	Merkel cell carcinoma Urothelial carcinoma Renal cell carcinoma
Durvalumab	Urothelial carcinoma Non-small cell lung cancer	Non-small cell lung cancer

## General aspects of toxicity

Both CTLA-4 and PD-1 inhibition negatively regulate T cell function. However, the biological and clinical impact on the immune system of CTLA-4 and PD-1 inhibition is different. In murine models, CTLA-4 deficiency has shown to cause lethal lymphoproliferative disorders [23], while PD-1 deficiency induces milder autoimmune diseases compatible with survival [24, 25]. These findings on murine models are paralleled by a different profile of clinical toxicity in patients receiving anti-CTLA-4 or anti-PD-1 agents. AEs are indeed more common and more severe with ipilimumab than with nivolumab or pembrolizumab [26].

Data from real-world studies showed that the most common irAEs were dermatologic, GI and endocrine disorders, with different frequencies depending on the cancer treated, the dose used and the type of patient [27]. GI and endocrine disorders were more frequent with anti-CTLA-4 agents, while myocarditis and autoimmune hepatitis were reported predominantly with anti-PD-1/PD-L1 drugs [27].

In phase 2 trials, 47-68% of patients treated with ipilimumab were observed to develop a diffuse maculopapular pruritic rush after approximately 3.6 weeks from treatment initiation [28, 29]. The lesions were histologically characterized by CD4 and Melan-A-specific CD8 T cells infiltrates extending into the dermis and the epidermis [30].

Immune-related hypophysitis are reported in 1–6% of patients receiving ipilimumab [28, 29, 31], and has been reported also with tremelimumab [32]. The symptoms occur after an average of 6 weeks of therapy initiation and consist of headache, nausea, vertigo, behavioral changes, diplopia and weakness. In case of hypophysitis, magnetic resonance imaging (MRI) shows enlargement or heterogeneity of the gland [31]. Blood tests typically show a low level of thyroid, adrenal and/or gonadal hormones.

Thyroid dysfunction occurs in up to 15% of patients and manifests as thyrotoxicosis, hypothyroidism, thyroid eye disease, painless thyroiditis and rarely severe forms of thyroid storm [33]. There have been also case reports of primary adrenal dysfunction with the use of these agents, with an incidence of 0.3–1.5% [33]. The development of new-onset insulin dependent diabetes has been described patients receiving anti-PD-1 or anti-PD-L1 antibodies, either as single agent or in combination with other cancer drugs [34, 35].

ICI-induced pneumonitis is not an uncommon AE with an overall incidence of 3–6%. Patients receiving combination therapy are more likely to manifest pneumonitis than patients undertaking monotherapy. Symptoms are not specific and can vary from dyspnea and cough to fever and chest pain [36].

Other rare irAEs include episcleritis/uveitis, sarcoid-like syndrome and neuropathies. Episcleritis/ uveitis occur in less than 1% of patients treated with ipilimumab, especially those affected also by diarrhea or colitis [37]. Typical symptoms are photophobia, dryness of the eyes and blurred vision with eye pain and occur after a median of 2 months from the initiation of therapy [37].

Diffuse lymphadenopathy and a sarcoid-like syndrome are reported with ipilimumab. This is proven by the presence of non-caseating granulomata in biopsy [38].

Transient peripheral neuropathies occur in less than 1% of patients which are usually minor and resolved spontaneously [39].

Myocarditis has emerged as rare but potentially lethal AE in patients treated with ICIs. Pharmacovigilance studies showed the highest fatality rate among all ICI-related AEs and it is most common in combination regimes [27, 40].

Immune arthritis, rheumatoid arthritis and polymyalgia rheumatica occurrence have also been reported after ICIs treatment [41, 42].

## GI toxicity

GI symptoms are the most common irAEs. They usually consist of abdominal pain, nausea, vomiting and diarrhea and are self-limiting in the majority of patients. Occasionally they can be severe and require specific treatments together with the withdrawal of the ICI agent [43].

The most common GI complication is diarrhea, which is more frequent with anti-CTLA-4 agents than with PD-1 inhibitors [44]. A meta-analysis by Wang et al. [44] showed an overall incidence of all-grade colitis of 13.6% in patients treated with a combination therapy of ipilimumab/nivolumab, of 9.1% in patients receiving ipilimumab and of 1.3% with anti-PD-1/PD-L1 monotherapy. An increased risk of GI AEs has been reported in patients taking nonsteroidal anti-inflammatory drugs [45]. Patients usually complain frequent non-bloody stools associated with urgency. Most of the times these manifestations are an expression of colonic inflammation, or enteritis in addition to colitis. However, patients may present with diarrhea caused by enteritis alone [46], which is not detected at endoscopy evaluation and can lead to small-bowel obstruction [47]. Endoscopic findings are nonspecific and characterized by mucosal edema, erythema and diffuse but shallow ulcers. However, the appearance of the mucosa may be normal [45]. The most common histopathologic pattern is crypt micro abscesses associated with apoptosis of crypt epithelial cells and atrophy of the crypt. Other histopathological features, such as lymphocytic colitis and inflammatory bowel disease-like patterns, are also possible [48]. Interestingly, the specific immune populations involved in the development of colitis have been reported to vary depending on the ICI-agent. Coutzac et al. [49] analyzed colon biopsies of 33 patients who developed immune-related colitis during anti-CTLA-4 or anti-PD-1 therapy. The immunohistochemistry of the samples showed that anti-PD-1 induced colitis was characterized by a predominant infiltration of CD8^+^ T cells in the lamina propria and epithelium, while anti-CTLA-4 colitis was associated with CD4^+^ T cells accumulation in the lamina propria with high tumor necrosis factor (TNF)-α secretion. Inflammation of the upper GI tract has been described with both anti-CTLA-4 and anti-PD-1. In a study conducted by Marthey et al. [45] duodenal biopsies performed in 10 patients receiving ipilimumab who presented endoscopic inflammation showed chronic duodenitis features (crypt distortion, villus shortening, lamina propria infiltration of eosinophils and mononuclear cells and Brunner’s gland hyperplasia). On the other hands gastric mucosa of 7 patients showed chronic gastritis without H. pylori and 1 patient presented granulomatous gastritis. A retrospective study by Collins et al. [50] on inflammatory GI disease induced by anti-PD-1 showed gastric biopsies with ulcerative and necrotic gastritis, neutrophils infiltration and intraepithelial lymphocytosis while in duodenal biopsies partial villus shortening, lymphocytosis of the epithelium and lamina propria infiltrated by lymphocytes and plasma-cells were observed. Moreover, a form of lymphocytic gastritis has been reported in patients with metastatic melanoma receiving pembrolizumab [51].

There is increasing clinical evidence that the gut microbiome has a profound impact on the tumor response to therapy and the development of GI toxicity. Specifically, Dubin et al. [52] analyzed in a prospective study the fecal samples of patients with metastatic melanoma before the initiation of ipilimumab. The authors showed that members of *Bacteroidetes* phylum were linked to worse cancer outcome and lower incidence of immune-related colitis, whereas *Firmicutes* were associated with better cancer response and enterocolitis occurrence [52]. Another prospective study by Chaput et al. [53] showed similar results. Specific bacteria have been associated with favorable antitumor outcome like *Faecalibacterium* spp., *Bifidobacterium* spp., *Bacteroides fragilis* and *Akkermansia muciphila* [54]. Furthermore, antibiotic therapy, which is a well-known microbiome disruptor, is reported to have a detrimental impact on the survival of patient undertaking anti-PD-1 [55].

**Figure 3.**
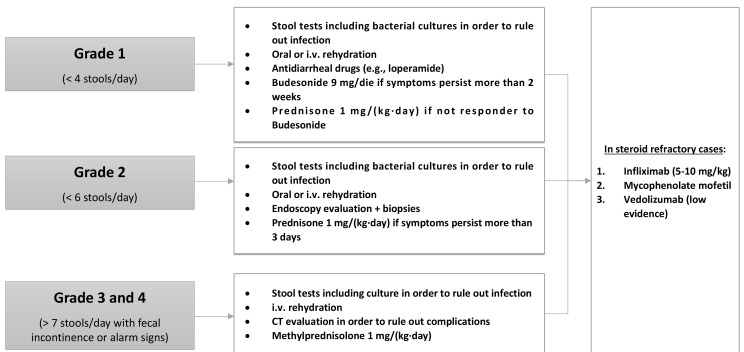
ICI-induced GI toxicity management. i.v.: intravenous

The management of GI AEs depends on the severity of the symptoms, which are classified into four grades (Figure 3).

Grade 1 diarrhea is characterized by a maximum of four stools per day. The first diagnostic step is to collect stool tests for bacterial pathogens, *Clostridium difficile* infection and other diarrhea-causing pathogens [47]. If infections are ruled out, mild diarrhea can be managed with oral or intravenous rehydration and antidiarrheal drugs such as loperamide [56]. If the symptoms persist for more than 2 weeks, patients should be treated with budesonide at 9 mg/die for at least 4 weeks before beginning tapering. If patients do not respond, a therapeutic switch to prednisone 1 mg/(kg·day) can be adopted [57].

Grade 2 diarrhea is characterized by a stool frequency of up to six stools per day. After infection is ruled out, endoscopic evaluation may be needed in patients with persistent symptoms (> 3 days) in order to establish the diagnosis of ICI-related colitis. While sigmoidoscopy with biopsies can be sufficient for patient evaluation, ileocolonoscopy may be required given the fact that patients may have enteritis without colitis. Nonetheless, the mucosa may be normal [46]. Treatment consists of fluid-replacement therapy with high dose corticosteroids. Oral prednisone [1 mg/(kg·day)] should be given when symptoms persist more than 3 days [57].

When patient present grade 3 or grade 4 diarrhea (more than seven stools per day associated with fecal incontinence), management requires hospitalization. These patients may present severe abdominal pain, fever, nausea, sepsis and rectal bleeding (alarm signs). A computed tomography (CT) scan exclusion of life-threatening complications such as bowel perforation, abscess formation and toxic megacolon before endoscopy evaluation is mandatory. The management includes intravenous fluids and, once infection is ruled out, intravenous methylprednisolone [1 mg/(kg·day)]. Patients who respond can receive oral prednisone at an equivalent dose after 3–5 days of clinical improvement with a gradual taper over 6–8 weeks. In refractory patients (persistent or progressive symptoms for over 3 days), treatment with infliximab (5–10 mg/kg) should be considered with mycofenolate mofetil as an alternative [57]. Preliminary data indicate that vedolizumab may be also used for the treatment of steroid-refractory cases or as a third-line therapy [58].

## Hepatotoxicity

ICI-induced liver injury is more common with CTLA-4 inhibitors compared to anti-PD-1 agents, with an incidence of 2–15% when patients are treated with monotherapy [59, 60]. On the other hand, combination therapy with ipilimumab and nivolumab is linked to an incidence of all-grade hepatotoxicity of 29% and severe liver injury of 17% [61]. Usually, patients are asymptomatic with a sporadic detection of transaminase and bilirubin increase at routine laboratory tests. Nonetheless some of the patients may experience fever, fatigue and jaundice [62].

When a liver biopsy is performed, lobular hepatitis with an infiltration of CD3^+^ or CD8^+^ T cells can be recognized. The histological pattern of immune-related hepatitis seems to be different between anti-CTLA-4 and anti-PD-1/anti-PD-L1 agents. Patients receiving anti-CTLA-4 agents predominantly showed a granulomatous form of hepatitis with fibrin ring deposits. Patients receiving anti-PD-1/anti-PD-L1 therapy had a tendency to develop a lobular, non-granulomatous form of hepatitis [63].

The risks factors that can predispose to ICI-induced hepatotoxicity are: underlying chronic liver disease [62], high dose of ipilimumab (10 mg/kg) [28], the use of CTLA-4 inhibitors or combination therapy [64, 65], the presence of other irAEs [66], liver metastases [62] and underlying autoimmune disorders such as thyroiditis or rheumatological disorders [67].

Patients treated with ICIs require monitoring liver enzymes levels at baseline and prior to each ICI infusion [56, 68, 69]. It is also important to rule out other causes of liver enzyme elevation. Basic testing should include markers of viral hepatitis [testing for hepatitis B virus (HBV), hepatitis C virus (HCV), hepatitis A virus (HAV), Hepatitis E virus (HEV), Epstein-Barr virus (EBV) and cytomegalovirus (CMV)], autoimmune hepatitis [testing for anti-nuclear antibodies (ANA), anti-mitochondrial antibody (AMA), anti-smoot muscle antibodies (ASMA) and liver-kidney microsomal antibodies (LKMA)], disease progression (investigated with ultrasonography and/or CT/MRI) and other drug-related adverse effect [56, 68, 69].

**Figure 4.**
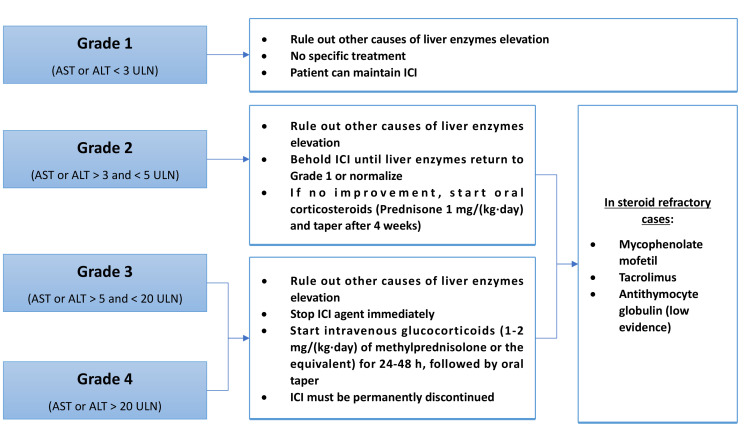
ICI-induced hepatotoxicity management

An elevation of liver enzyme more than 2 times the upper limit of normal (ULN) needs further evaluation and its management depends on the grade of liver injury (Figure 4).

Grade 1 hepatitis [aspartate aminotransferase (AST) or alanine aminotransferase (ALT) < 3 ULN] do not need any specific treatment and the patient may continue their prescribed agent [56, 68, 69].

In case of grade 2 hepatitis (AST or ALT > 3 ULN), ICIs administration should be suspended until enzymes return to grade 1 level or normalize. If there is no improvement, oral corticosteroids [0.5–1 mg/(kg·day) of prednisone] can be given and tapering should be initiated after 4 weeks [56, 68, 69]. If grade 2 hepatotoxicity persists in patients with negative viral hepatitis despite 3 days of steroid therapy, liver biopsy is recommended in order to confirm other etiologies of liver injury [56].

For grades 3 (AST or ALT > 5 ULN) and 4 (AST or ALT > 20 ULN), the patient should be treated with intravenous glucocorticoids [1–2 mg/(kg·day) of methylprednisolone] for 24 to 48 h, followed by oral steroid taper. ICI agent suspension is mandatory [56, 68, 69]. In steroid-refractory cases, immunosuppressive agents such as mycophenolate mofetil and tacrolimus can be used. On the other hand, Infliximab should not be used in this setting because of the risk of hepatotoxicity associated with anti-TNF-α [69]. It has also been reported a successful treatment with antithymocyte globulin in a patient with ICI-induced hepatitis refractory to standard therapy [70]. Grade 3 and 4 hepatitis require a permanent withdrawal of the ICIs agent [56, 68, 69].

## Pancreatic toxicity

The incidence of ICI-induced pancreatic injury is low (about 2.7% of treated patients) and usually characterized by an asymptomatic elevation of serum lipase with normal imaging findings of the pancreas [71, 72]. As for the others irAEs, pancreatitis is reported to be more frequent in patients treated with anti-CTLA-4 agents than anti-PD-1/PD-L1 (3.98% *vs.* 0.94%) and in patients receiving a combination therapy [71]. Given the vague clinical significance of isolated elevated lipase levels, routine monitoring of pancreatic enzymes is not recommended [73]. Acute pancreatitis is diagnosed when patient present acute onset of persistent, severe epigastric pain associated with an elevation of lipase/amylase higher than three times ULN and/or characteristic findings on abdominal imaging [57]. In a retrospective study, 39% of patients with grade 3 lipase elevation or higher presented typical symptoms of acute pancreatitis. The patients with clinical symptoms were more likely to present abnormal CT findings [72]. Emerging evidence indicate that intravenous fluid administration should be given to all patients with grade 3 or higher lipase levels, even without clinical symptoms. This approach may lower the risk of long-term adverse outcomes [72]. In the absence of symptoms, corticosteroid treatment should be avoided [73]. Other than acute pancreatitis, other forms of pancreatic injury has been reported such as exocrine insufficiency [74] and new-onset diabetes [35].

## Conclusion

In summary, ICIs improved the outcome of an increasing number of advanced cancers, but not without drawbacks. IrAEs are not rare but most frequently mild in grade and do not affect patient’s ability to receive further immunotherapy. Nonetheless, early recognition of severe cases and proper management is crucial to minimize life-threatening complications of this antineoplastic therapy. Severe toxicities need to be treated aggressively with corticosteroids or immunosuppressive agents in addition to at least a temporary withdrawal of the immunotherapy. In most cases that require a specific treatment, reintroduction of the ICI agent must be discussed in a multidisciplinary context, as relapses are common. In most severe toxicities ICI therapy must be permanently interrupted.

## Abbreviations

AEs: adverse events
ALT: alanine aminotransferase
APC: antigen-presenting cell
AST: aspartate aminotransferase
CT: computed tomography
CTLA-4: cytotoxic T-lymphocyte antigen 4
GI: gastrointestinal
ICIs: immune checkpoint inhibitors
IrAEs: immune-related adverse events
PD-1: programmed cell death 1
PD-L1: programmed death ligand 1
TCR: T cell receptor
ULN: upper limit of normal

## Declarations

### Copyright

© The Author(s) 2021.
